# Cefiderocol activity against multidrug-resistant isolates from the PERSEUS study: relationship between cefiderocol minimum inhibitory concentration and clinical outcomes

**DOI:** 10.1093/jac/dkag164

**Published:** 2026-05-26

**Authors:** Rafael Cantón, María García-Castillo, Marta Hernández-García, Miquel Àngel Sastre-Femenía, Carla López-Causapé, Patricia Ruiz-Garbajosa, Lucía García Labrador, Christopher Longshaw, Antonio Oliver

**Affiliations:** Servicio de Microbiología, Hospital Universitario Ramón y Cajal and Instituto Ramón y Cajal de Investigación Sanitaria (IRYCIS), Madrid, Spain; CIBER de Enfermedades Infecciosas (CIBERINFEC), Instituto de Salud Carlos III, Madrid, Spain; Servicio de Microbiología, Hospital Universitario Ramón y Cajal and Instituto Ramón y Cajal de Investigación Sanitaria (IRYCIS), Madrid, Spain; CIBER de Enfermedades Infecciosas (CIBERINFEC), Instituto de Salud Carlos III, Madrid, Spain; Servicio de Microbiología, Hospital Universitario Ramón y Cajal and Instituto Ramón y Cajal de Investigación Sanitaria (IRYCIS), Madrid, Spain; CIBER de Enfermedades Infecciosas (CIBERINFEC), Instituto de Salud Carlos III, Madrid, Spain; CIBER de Enfermedades Infecciosas (CIBERINFEC), Instituto de Salud Carlos III, Madrid, Spain; Servicio de Microbiología, Hospital Universitario Son Espases, Instituto de Investigación Sanitaria Illes Balears (IdISBa), Palma de Mallorca, Spain; CIBER de Enfermedades Infecciosas (CIBERINFEC), Instituto de Salud Carlos III, Madrid, Spain; Servicio de Microbiología, Hospital Universitario Son Espases, Instituto de Investigación Sanitaria Illes Balears (IdISBa), Palma de Mallorca, Spain; Servicio de Microbiología, Hospital Universitario Ramón y Cajal and Instituto Ramón y Cajal de Investigación Sanitaria (IRYCIS), Madrid, Spain; CIBER de Enfermedades Infecciosas (CIBERINFEC), Instituto de Salud Carlos III, Madrid, Spain; Medical Affairs, Shionogi S.L.U., Madrid, Spain; Medical Affairs, Shionogi B.V., London, UK; CIBER de Enfermedades Infecciosas (CIBERINFEC), Instituto de Salud Carlos III, Madrid, Spain; Servicio de Microbiología, Hospital Universitario Son Espases, Instituto de Investigación Sanitaria Illes Balears (IdISBa), Palma de Mallorca, Spain

## Abstract

**Objective:**

The PERSEUS study was a retrospective analysis of cefiderocol use under the compassionate use and early access programmes in Spain for the treatment of MDR Gram-negative infections. We present the microbiological evaluation of 57 isolates collected from this study, along with their correlation to clinical outcomes.

**Methods:**

Cefiderocol susceptibility testing was performed using standard broth microdilution (BMD) in iron-depleted media, commercial BMD panels (UMIC^®^, ComASP^®^), gradient MIC strips, and disk diffusion. Results were interpreted according to EUCAST 2024 criteria. WGS was used to characterize β-lactamase genes and mutations in genes implicated in cefiderocol resistance. Patients’ clinical charts were reviewed.

**Results:**

Most isolates were identified as *Pseudomonas aeruginosa* (72%). Cefiderocol demonstrated an overall susceptibility rate of 86%, outperforming most comparators except colistin. Commercial BMD devices demonstrated high concordance with the reference method, supporting their potential value for routine use. Molecular analysis revealed a high prevalence of class B metallo-β-lactamases, as well as mutations in iron transport genes, among resistant isolates. Notably, clinical cure was achieved even in patients infected with cefiderocol-resistant isolates treated with cefiderocol monotherapy, suggesting that some putative resistance mechanisms affect the MIC, without necessarily leading to clinical failure.

**Conclusions:**

Positive clinical outcomes in patients infected by cefiderocol-resistant isolates by EUCAST breakpoints highlight the need for standardized susceptibility testing, as well as deeper genotype-phenotype correlation studies. Cefiderocol remains a promising option for treating MDR Gram-negative infections, especially those caused by *P. aeruginosa*. Its adoption in clinical practice should be supported by validated testing methods and ongoing surveillance.

## Introduction

The global increase in antimicrobial resistance (AMR), particularly among Gram-negative pathogens, remains an urgent threat to public health and patient outcomes.^1,2^ One of the most significant challenges is the growing prevalence of third-generation cephalosporin- and carbapenem-resistant Enterobacterales and carbapenem-resistant *Pseudomonas aeruginosa*. The WHO has classified these organisms as critical as well as high priority pathogens due to their high risk of morbidity, mortality, and limited therapeutic options available.^1^

Cefiderocol, a novel siderophore cephalosporin approved in Europe in 2020, was developed to address the therapeutic gap against MDR Gram-negative pathogens.^3,4^ The conjugation of a cephalosporin core to an iron-binding siderophore domain enables its active transport across the bacterial outer membrane after iron chelation.^3,5–7^ Also, this unique structure confers enhanced stability to hydrolysis by a broad spectrum of β-lactamases, including carbapenemases, and renders it a poor substrate for MDR efflux pumps.^3,5–7^ Preclinical, clinical, and real-world data support cefiderocol’s *in vitro* and *in vivo* activity against many MDR Gram-negative bacteria, providing an adequate therapeutic option for patients with limited alternatives.^3,8–11^ Susceptibility rates are generally higher than those of other commercially available β-lactams (with and without β-lactamase inhibitors), and the development of resistance often requires a combination of multiple mechanisms, including the production of one or more β-lactamases and the inactivation of proteins involved in siderophore-mediated antibiotic uptake.^11–19^

Despite its potential, the clinical use of cefiderocol is hindered by practical limitations, particularly regarding susceptibility testing. Accurate and reproducible assessment of cefiderocol activity in routine microbiology laboratories is not yet easily achieved, resulting in potential overestimation of resistance, which may lead to inappropriate antimicrobial selection, delayed initiation of effective therapy, and poorer clinical outcomes, particularly in critically ill patients with limited treatment options. Broth microdilution (BMD) in iron-depleted media is the reference method according to the EUCAST criteria, but the media preparation is complex and, therefore, difficult to implement as routine testing in microbiology laboratories.^20–22^ Alternatively, EUCAST proposes using the quantitative disk diffusion (DD) method. However, the area of technical uncertainty (ATU) becomes a limitation when interpreting the results.^23,24^ ComASP^®^ or UMIC^®^ commercial devices have shown promising results, but they are still under evaluation by EUCAST and may require complementary testing to guide informed decision making.^20,21,23,25,26^

In 2025, de la Torre-Cisneros *et al.* published the results of the PERSEUS study: an observational retrospective study aimed at assessing the efficacy and safety of cefiderocol in 261 hospitalized patients with serious Gram-negative bacterial infections (excluding *Acinetobacter* spp. infections), from the compassionate use and early access programmes (EAP) in Spain.^27,28^ The overall clinical cure rate was 80.5%, and the 28-day mortality rate was 21.5%; whereas in the subgroup of patients with *P. aeruginosa* infection, these rates were 84.5% and 17.2%, respectively. Prior antibiotic treatment and mechanical ventilation were identified as negative predictors of clinical cure. Adverse events occurred in seven patients, one of which resulted in a fatal case of toxic epidermal necrolysis. However, this publication did not include an analysis of the microbiological samples collected at baseline.

As PERSEUS was a retrospective study with no predefined instructions on sample storage, only a small proportion of the 261 isolates were available for retesting in a central laboratory and microbiological characterization. The current study examined the susceptibility of the available isolates (*n* = 57) from the PERSEUS study to cefiderocol and other antibiotics, as well as the performance of commercially available *in vitro* diagnostic tests for evaluating cefiderocol activity compared with the reference BMD method. WGS was used to identify acquired β-lactamases and other molecular determinants implicated in cefiderocol resistance in these isolates. Additionally, the study explored the relationship between cefiderocol MIC, the presence of putative resistance-associated genes or mutations, and clinical outcomes.

## Materials and methods

### Bacterial isolates

A total of 57 isolates were included for testing at the central laboratory: 43 *Pseudomonas* spp. (41 *P. aeruginosa*, one *P. mosselii*, one *P. nitroreducens*), six *Klebsiella pneumoniae*, two *Enterobacter hormaechei*, and six others (three *Burkholderia cepacia* complex, and three *Stenotrophomonas maltophilia*).

### Strains isolation and identification

#### Bacterial identification

Bacterial identification was performed with MALDI-TOF MS (Bruker, Billerica, MA, USA) and confirmed by ribosomal multilocus sequence typing (rMLST).

#### Antimicrobial susceptibility testing

Cefiderocol susceptibility was evaluated by different commercial methods including (ⅰ) BMD methods: UMIC^®^ Cefiderocol (Bruker) and ComASP^®^ Cefiderocol 0.008–128 mg/L (Liofilchem, Roseto degli Abruzzi, Italy); (ⅱ) MIC Test Strip^TM^ Cefiderocol 0.016–256 mg/L (Liofilchem) (only for *P. aeruginosa*), and (ⅲ) DD (Oxoid, ThermoFisher, Waltham, MA, USA). Results were compared with those obtained by the standard BMD method according to International Standard ISO 20776-1 guidelines, using 96-well microtiter plates, and following EUCAST recommendations.^22,29^ This susceptibility testing was performed in the central laboratory (Hospital Universitario Ramón y Cajal, Madrid). In addition, this laboratory performed susceptibility testing to other antibiotics using BMD panels manufactured by Sensititre (EUMDROXF panel, ThermoFisher). The antimicrobials and the tested concentration ranges are depicted in Table S1 (available as Supplementary data at *JAC* Online). The obtained results were interpreted according to EUCAST-2024 or CLSI criteria.^30,31^ Categorical agreement (CA; ≥90%), essential agreement (EA; ≥90%), and bias (−30%/30%) were calculated relative to the reference method following ISO recommendations.^29^

### Whole genome sequencing and molecular characterization

Genomic DNA was obtained with a commercially available extraction kit (High Pure PCR Template Preparation Kit, Roche Diagnostics Ltd., Burgess Hill, UK), and indexed paired-end libraries were prepared with the Illumina DNA Prep Library Preparation Kit (Illumina Inc., San Diego, CA, USA) and sequenced on an Illumina NovaSeq 6000 platform (Illumina Inc.).

Paired-end short reads were *de novo* assembled with SPAdes v3.15.5 using the careful option.^32^  *De novo* assemblies were used to confirm the bacterial species by using rMLST (available at https://pubmlst.org/species-id) as well as to determine the ST using the MLST open-source software (https://github.com/tseemann/mlst), which uses the publicly available PubMLST typing schemes and database (https://pubmlst.org/; db v2.23.0). To explore the presence of horizontally acquired antibiotic resistance genes, we used the ResFinder analysis tool and its manually curated database (v.2.4.0).^33^

In addition, a variant calling analysis was performed on the 41 *P. aeruginosa* isolates, using the Snippy software v4.6.0. (https://github.com/tseemann/snippy) and the PAO1 reference genome (NC_002516.2), in order to identify mutations in 48 chromosomal genes related to antibiotic resistance and in a set of iron-related genes (*pfeA, fptA, piuA, piuD, piuC, cirA, pirR, pirS, pirA*) that have been associated with cefiderocol resistance.^14,34^ Large chromosomal deletions were investigated with the visualization tool SeqMonk v1.47.2. (https://www.bioinformatics.babraham.ac.uk/projects/seqmonk/). For Enterobacterales, reference genomes used were: NZ_CANDXJ010000001-ST147—*K. pneumoniae*, NZ_JBBFTN010000001-ST512—*K. pneumoniae*, NZ_JBBFTN010000001-ST88—*E. hormaechei*. In Enterobacterales isolates, the following genes previously related to cefiderocol resistance were analysed: *fiu, cirA, exbB, exbD, tonB, ftsI, baeS/R, ompR/envZ, ompC,* and *ompF*.

## Results

### Susceptibility profile of isolates to cefiderocol and comparators

Overall, 57 Gram-negative isolates derived from the PERSEUS study^27,28^ were included in this research. The identification by rMLST confirmed that 43 belonged to *Pseudomonas* spp. (41 *P. aeruginosa*, one *P. mosselii*, and one *P. nitroreducens*), while eight belonged to the Enterobacterales family (six *K. pneumoniae* and two *E. hormaechei*). Additionally, the sample comprised two *Burkholderia contaminans*, one *B. dolosa*, and three *S. maltophilia*.

According to the reference BMD method, cefiderocol demonstrated excellent *in vitro* activity among the available PERSEUS isolates. The overall MIC_50_ and MIC_90_ were 0.5 mg/L and 4 mg/L, respectively (*n* = 57), and susceptibility rate was 86% (49/57) (Table 1). These isolates were highly resistant to all other tested antibiotics, except for colistin, with a susceptibility rate of 93% (53/57). Susceptibility rates for ceftazidime-avibactam, ceftolozane-tazobactam, and imipenem-relebactam were 22.8% (13/57), 19.3% (11/57), and 10.5% (6/57), respectively (Table 1). When considering only Enterobacterales and *Pseudomonas* spp., susceptibility rate to cefiderocol was 84.3% (43/51) (Table S2). However, it reached 92.7% (38/41) among *P. aeruginosa* isolates (Table 2, Table S3).

**Table 1.** Cumulative MIC distribution as a percentage and susceptibility according to EUCAST breakpoints for the 57 isolates characterized from PERSEUS study

Antimicrobials	MIC (mg/L)												EUCAST (%)		
	0.06 or ≤0.06	0.12 or ≤0.12	0.25 or ≤0.25	0.5	1	2 or ≤2	4	8 or >4	16 or >8	>16 or 32	64	>64	S	I	R
**Aztreonam**	—	—	—	—	0.0	1.8	3.5	19.3	26.3	47.4	***100.0***	—	1.8	24.6	57.4
**Piperacillin/tazobactam**	—	—	—	—	—	—	0.0	1.8	1.8	7.0	***100.0***	—	1.8	0.0	98.2
**Cefepime**	—	—	—	—	1.8	1.8	1.8	1.8	17.5	***100.0***	—	—	1.8	0.0	98.2
**Ceftazidime-avibactam**	—	—	0.0	0.0	5.3	5.3	7.0	22.8	38.6	***100.0***	—	—	22.8	0.0	77.2
**Ceftolozane-tazobactam**	—	—	0.0	1.8	1.8	8.8	19.3	29.8	***100.0***	—	—	—	19.3	0.0	80.7
**Imipenem**	—	—	—	—	1.8	1.8	5.3	10.5	***100.0***	—	—	—	1.8	3.5	94.7
**Imipenem-relebactam**	0.0	0.0	0.0	1.8	5.3	10.5	29.8	40.4	***100.0***	—	—	—	10.5	0.0	89.5
**Meropenem**	—	0.0	0.0	1.8	1.8	1.8	1.8	7.0	31.6	***100.0***	—	—	1.8	5.3	92.9
**Meropenem-vaborbactam**	0.0	0.0	0.0	1.8	1.8	3.5	7.0	17.5	38.6	***100.0***	—	—	17.5	0.0	82.5
**Tigecycline**	—	—	—	10.5	15.8	***100.0***	—	—	—	—	—	—	—	—	—
**Eravacycline**	0.0	0.0	5.3	7.0	***100.0***	—	—	—	—	—	—	—	—	—	—
**Amikacin**	—	—	—	—	—	10.5	24.6	40.4	**63**.**2**	66.7	*100.0*	—	63.2	0.0	36.8
**Tobramycin**	—	—	—	15.8	22.8	26.3	28.1	***100.0***	—	—	—	—	26.3	0.0	73.7
**Fosfomycin**	—	—	—	—	—	—	—	0.0	8.8	15.8	26.3	***100.0***	—	—	—
**Colistin**	—	—	—	24.6	**56**.**1**	87.7	93.0	93.0	*94.7*	100.0	—	—	93.0	0.0	7.0
**Cefiderocol**	—	8.8	29.8	**54**.**4**	77.1	86.0	*91.2*	96.5	98.3	100.0	—	—	86.0	0.0	14

Numbers in bold correspond to MIC_50_ and italic numbers to MIC_90_.

**Table 2.** Cumulative MIC distribution as a percentage for the 41 *P. aeruginosa* isolates

Antimicrobials	MIC (mg/L)												EUCAST (%)		
	0.06 or ≤0.06	0.12 or ≤0.12	0.25 or ≤0.25	0.5	1	2 or ≤2	4	8 or >4	16 or >8	>16 or 32	64	>64	S	I	R
**Aztreonam**	—	—	—	—	0.0	0.0	2.4	22.0	31.7	**58**.**5**	*100.0*	—	2.4	29.3	68.3
**Piperacillin/tazobactam**	—	—	—	—	—	—	0.0	2.4	2.4	9.8	***100.0***	—	2.4	0.0	97.6
**Cefepime**	—	—	—	—	2.4	2.4	2.4	2.4	19.5	***100.0***	*—*	—	2.4	0.0	97.6
**Ceftazidime-avibactam**	—	—	0.0	0.0	2.4	2.4	4.9	19.5	31.7	***100.0***	—	—	19.5	0.0	80.5
**Ceftolozane-tazobactam**	—	—	0.0	2.4	2.4	12.2	26.8	36.6	***100.0***	—	—	—	26.8	0.0	73.2
**Imipenem**	—	—	—	—	2.4	2.4	7.3	9.8	***100.0***	—	—	—	2.4	4.9	92.7
**Imipenem-relebactam**	0.0	0.0	0.0	2.4	4.9	9.8	34.1	39.0	***100.0***	—	—	—	9.8	0.0	90.2
**Meropenem**	—	0.0	0.0	2.4	2.4	2.4	2.4	4.9	31.7	***100.0***	—	—	2.4	2.4	95.1
**Meropenem-vaborbactam**	0.0	0.0	0.0	2.4	2.4	2.4	4.9	14.6	41.5	***100.0***	—	—	14.6	0.0	85.4
**Tigecycline**	—	—	—	2.4	2.4	***100.0***	—	—	—	—	—	—	—	—	—
**Eravacycline**	0.0	0.0	0.0	2.4	***100.0***	—	—	—	—	—	—	—	—	—	—
**Amikacin**	—	—	—	—	—	9.8	24.4	46.3	**70**.**7**	73.2	*100.0*	—	70.7	0.0	29.3
**Tobramycin**	—	—	—	22.0	29.3	34.1	34.1	***100.0***	—	—	—	—	34.2	0.0	65.9
**Fosfomycin**	—	—	—	—	—	—	—	0.0	9.8	17.1	22.0	***100.0***	—	—	—
**Colistin**	—	—	—	12.2	**51**.**2**	*92.7*	97.6	97.6	100.0	100.0	—	—	97.6	0.0	2.4
**Cefiderocol**	—	4.9	26.8	**56**.**1**	82.9	*92.7*	92.7	97.6	97.6	100.0	0	0	92.7	0.0	7.3

Numbers in bold correspond to MIC_50_ and italic numbers to MIC_90_.

There were eight cefiderocol-resistant isolates according to EUCAST breakpoints, which comprised: three *K. pneumoniae* (two belonging to ST147 and one to ST512), one *E. hormaechei* (ST88), three *P. aeruginosa* (ST253, ST309, and ST2056-1LV), and one *P. nitroreducens*. MICs to cefiderocol in these resistant isolates ranged from 4 to 32 mg/L, with only two isolates presenting resistance according to CLSI breakpoints (one *K. pneumoniae* with an MIC of 16 mg/L and one *P. aeruginosa* with an MIC of 32 mg/L).

### Molecular characterization of acquired β-lactamases

WGS analysis revealed that 54.4% (31/57) of the isolates encoded at least one acquired β-lactamase. Their presence was observed in 100% (8/8) of Enterobacterales isolates, 51.2% (21/41) of *P. aeruginosa* isolates, and 100% (2/2) of *P. mosselii* and *P. nitroreducens* isolates. No acquired β-lactamases were identified among *Burkholderia* spp. and *S. maltophilia* isolates, although the latter carried two chromosomally encoded β-lactamases: L1 carbapenemase and L2 cephalosporinase.^35^ Full details on β-lactamases and resistance determinants to β-lactams and other antibiotics derived from WGS are provided in Table S4.

According to Ambler classification, we identified a predominance of class B metallo-β-lactamases (MBL), which were detected in 71% (22/31) of isolates that had acquired a β-lactamase. Class A, C, and D β-lactamases were identified in 32.2%, 9.7%, and 45.2% of cases, respectively. Class B β-lactamases were particularly prevalent among *Pseudomonas* spp. isolates (78.3%, 18/23) (Table S5).

Among acquired β-lactamase-producing *Pseudomonas* spp. isolates (*n*=23), the most prevalent were VIM-type (VIM-2 in 47.8% and VIM-1 in 17.4% of isolates), followed by OXA-2 (26.1%) and ESBL derivatives OXA-1372 (aa148Δ6) in 4.4%, and OXA-210 (Y158C) in 4.4% of isolates. Only 16.3% isolates (7/43) encoded more than one acquired β-lactamase. Six isolates presented two β-lactamases (VIM-2, OXA-2 [*n* = 2]; CARB-4 [E203K], VIM-1 [*n* = 2]; and VIM-20, OXA-2 [*n* = 2]) and one isolate had four β-lactamases (CARB-4 [E203K], OXA-4, IMP-15, FOX-4 [H220Y, V233A]).

Despite the limited number of Enterobacterales isolates included in this study, we observed a trend towards multiple β-lactamase production (75%, 6/8), with class A ESBL combined with different carbapenemases (mainly OXA-48, NDM, or VIM) being the most frequent pattern. Three isolates presented two β-lactamases (CTX-M-15, OXA-48 [*n* = 1] and CTX-M-15, NDM-1 [*n* = 2]), two had three β-lactamases (KPC-3, TEM-1A, OXA-9 [W112X] and DHA-1, TEM-1B, OXA-1), and one presented six β-lactamases (CMY-4, NDM-1, TEM-1B, SHV-12, VIM-1, OXA-1).

### Assessing commercially available cefiderocol susceptibility assays

**Figure 1.**
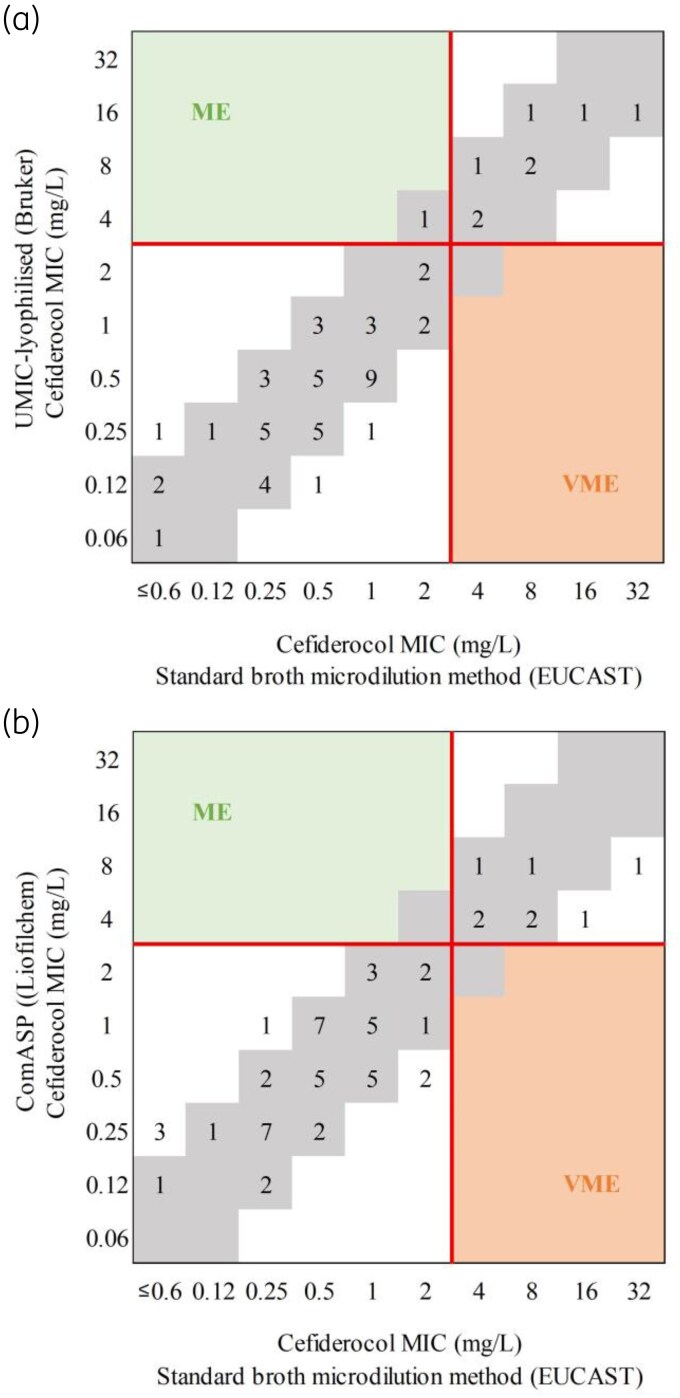
Correlation between MICs of cefiderocol obtained using (a) UMIC-lyophilised (Bruker) (b) ComAsp (Liofilchem) compared with the standard BMD method according to International Standard ISO 20776-1 guidelines and following EUCAST recommendations (*n* = 57). MICs corresponding to essential agreement are depicted in grey, major error (ME) in green, and very major error (VME) in orange.

We tested all 57 isolates for cefiderocol susceptibility using all commercially available methods, including DD and BMD methods, and compared the results to the reference BMD method, according to EUCAST guidelines.^22^ Regarding the DD method, although the CA was 96.5%, we detected two very major errors, involving one *P. aeruginosa* and one *P. nitroreducens* isolate. Commercially available BMD panels (UMIC^®^ and ComASP^®^) showed a very good correlation with the reference method, with CA and EA values >90% (UMIC^®^-lyophilised: CA 98.2%, EA 96.5%, bias −22.8%; ComASP^®^: CA 100%, EA 91.2%, bias −3.5%). No very major errors were detected, and only one major error was identified with the UMIC^®^-lyophilised test (Figure 1, Table S6). These tests are manufactured following ISO 20776-1 standards.^29^ Gradient MIC strips were only tested with *Pseudomonas* spp. isolates, since they have only been validated for evaluating cefiderocol susceptibility in that species according to the manufacturer. Notable limitations were identified: while these tests exhibited a high CA with the reference method (97.6%), EA was markedly lower at 24.4%, and the test showed a negative bias of −85.4%, with gradient strips reporting lower MICs than the reference method. Two very major errors were detected among *Pseudomonas* spp. isolates, involving one *P. aeruginosa* and one *P. nitroreducens.* These were the same isolates that led to very mayor errors with the DD method.

### Molecular determinants associated with cefiderocol resistance

Regarding cefiderocol-resistant isolates (Enterobacterales [*n* = 4], *Pseudomonas* spp. [*n* = 4]), all isolates (8/8) presented at least one acquired β-lactamase, with class B NDM-1 being the most prevalent one (37.5%, 3/8). In contrast to other studies, mutations in penicillin-binding protein 3 (PBP3) were absent among the resistant isolates in this collection.^12–14^ Meanwhile, 75% (6/8) of these isolates exhibited mutations in genes involved in cefiderocol transport across the plasma membrane, primarily affecting iron transport systems (*cirA, tonB, piuA,* and *pirR*). These mutations may contribute to resistance to cefiderocol but not to other β-lactams. Inactivating mutations were detected in two *P. aeruginosa* isolates (*pirR*-nt394Δ1 and *piuA*-nt1Δ193) and two of the *K. pneumoniae* (*cirA*-V297fs) resistant isolates both belonging to ST147 (Table 3, Table S4).

**Table 3.** Characteristics of cefiderocol-resistant isolates, patients infected by those isolates, and clinical outcomes

Microorganism	FDC MIC (mg/L)	Acquired resistance genes		Primary infection site	Secondary bloodstream infection	Patient in ICU while receiving cefiderocol	Mechanical ventilation at baseline	Extracorporeal membrane oxygenation (ECMO)
		Acquired beta-lactamase	Exclusive mutations for cefiderocol resistance					
*E. hormaechei*	4	CMY-4, ACT-17, NDM-1, TEM-1B, SHV-12, VIM-1, OXA-1	*ompF*-A137fs	Respiratory	No	Yes	Yes	Yes
*K. pneumoniae*	4	CTX-M-15, NDM-1	*cirA*-V297fs	Intra-abdominal	No	Yes	Yes	No
*P. nitroreducens*	4	VIM-1		Urine	No	No	No	No
*K. pneumoniae*	8	CTX-M-15, NDM-1	*cirA*-V297fs	Respiratory	Yes	Yes	Yes	No
*P. aeruginosa*	8	OXA-2 (aa148Δ6)	*piuA*-nt1Δ193	Intra-abdominal	No	Yes	No	No
*P. aeruginosa*	8	OXA-2		Skin and Soft Tissue	No	No	No	No
*K. pneumoniae*	16	KPC-3, TEM-1A, OXA-9 (W112X)	*tonB-*E672A	Respiratory	No	No	No	No
*P. aeruginosa*	32	GES-7	*pirR*-nt394Δ1	Respiratory	No	No	No	No

Microorganism	Renal replacement therapy (RRT)	Patient previously colonized by this pathogen	Prior Gram-negative antibiotic treatment for the reference infection	Number of prior Gram-negative antibiotics	Prior antibiotics	Cefiderocol initiated as a combination regimen	Susceptible antibiotic added to cefiderocol during the course of treatment	Septic shock	Clinical cure	Survival at day 28
*E. hormaechei*	Yes	No	Yes	3	Meropenem, amikacin, colistin	Yes	Amikacin	Yes	Yes	No
*K. pneumoniae*	Yes	Yes	Yes	8	Colistin, amikacin, aztreonam, ceftazidime-avibactam, ciprofloxacin, meropenem, trimethoprim/sulfamethoxazole, tigecycline	No	No	No	No	No
*P. nitroreducens*	No	No	No	0	—	No	No	No	Yes	Yes
*K. pneumoniae*	Yes	Yes	Yes	4	Levofloxacin, amikacin, trimethoprim/sulfamethoxazole, colistin	Yes	No	Yes	Yes	No
*P. aeruginosa*	No	Yes	Yes	2	Meropenem, aztreonam	No	No	No	Yes	Yes
*P. aeruginosa*	No	No	Yes	3	Meropenem, amikacin, tigecycline	Yes	No	No	Yes	Yes
*K. pneumoniae*	No	No	Yes	3	Aztreonam, and aztreonam and ceftazidime-avibactam,	No	No	Yes	Yes	Yes
*P. aeruginosa*	No	No	No	0	—	No	Colistin	No	Yes	Yes

### Correlation between cefiderocol susceptibility status and clinical cure

**Figure 2.**
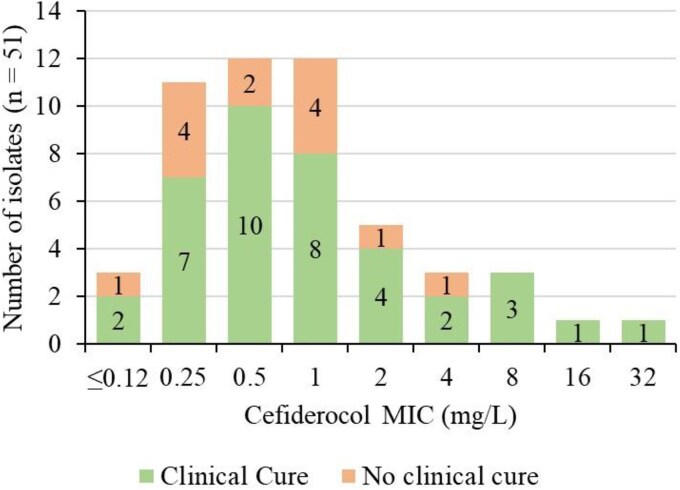
MIC distribution for cefiderocol for patients included in the primary analysis (*n* = 51). MICs were obtained using the standard BMD method according to International Standard ISO 20776-1 guidelines and following EUCAST recommendations.

Of the 57 isolates identified in this study, only 51 corresponded to patients who met the eligibility criteria for inclusion in the primary clinical analysis of the PERSEUS study.^27,28^ In the current subset of patients, the clinical cure rate was 74.5% (38/51), and the 28-day mortality rate was 23.5% (12/51). Among patients infected with *P. aeruginosa*, clinical cure was observed in 26/35 (74.3%) of them, while among patients infected with Enterobacterales, clinical cure was achieved in 7/8 (87.5%). Mortality rate at Day 28 was 17.1% (6/35) and 37.5% (3/8) in *P. aeruginosa* and Enterobacterales infections, respectively. For the patients who achieved clinical cure, cefiderocol MICs ranged from <0.12 to 32 mg/L; while for the patients who did not achieve clinical cure, they ranged from <0.12 to 4 mg/L (Figure 2), suggesting that resistance according to current breakpoints was not predictive of clinical failure in these cases.

As for patients infected with cefiderocol-resistant isolates (MIC >2 mg/L; *n* = 8), only one did not achieve clinical cure. The remaining seven patients recovered from the infection despite displaying MICs to cefiderocol ranging from 4 to 32 mg/L (Table 3). Moreover, all these isolates encoded at least one β-lactamase, and 5/7 showed mutations specifically associated with cefiderocol resistance. Three patients started cefiderocol as part of a combination regimen alongside another antibiotic to which the isolate was susceptible; and for one patient, intravenous colistin was added while on treatment with cefiderocol. In another case, amikacin was continued for 3 days after starting cefiderocol treatment to complete the 5-day amikacin treatment course previously prescribed. Information regarding the primary site of infection, patient hospital management, and prior antibiotic treatment is summarized in Table 3.

## Discussion

Cefiderocol has emerged as a promising alternative treatment for infections caused by MDR Gram-negative pathogens, particularly *P. aeruginosa*. The isolates included in this study presented a high selection bias, since they were not consecutive isolates but originated from a Shionogi compassionate use programme and an EAP in Spain, where cefiderocol was requested only when no other therapeutic options were available due to resistance or potential adverse events for life-threatening infections caused by Gram-negative bacteria. Evidencing this, 80.4% (41/51) of patients had received previous antibiotic treatment for the infection with the respective isolated Gram-negative bacteria before starting cefiderocol treatment.

In our study, cefiderocol demonstrated a good susceptibility profile among the PERSEUS isolates included in this analysis, even when compared with newer combinations of β-lactams plus β-lactamase inhibitors, proving to be a reliable alternative for treating infections by difficult-to-treat resistance microorganisms, particularly those caused by *P. aeruginosa*. Cefiderocol susceptibility rate (86%) was higher than for all other tested antibiotics, including ceftazidime-avibactam (22.8%), ceftolozane-tazobactam (19.3%), or imipenem-relebactam (10.5%) (Table 1). Only colistin showed also a high susceptibility rate (93%). We observed no cross-resistance with other β-lactams, which supports cefiderocol’s unique uptake mechanism as a siderophore cephalosporin and its increased stability to β-lactamase hydrolysis. This finding is consistent with multiple reports demonstrating the potent *in vitro* activity of cefiderocol against MDR Gram-negative bacteria, including carbapenem-resistant isolates.^10,11,13,15,36^ Notably, while colistin usually shows high *in vitro* susceptibility rates, its use is limited due to the significant risk of nephrotoxicity and neurotoxicity.^37,38^

Molecular characterization of PERSEUS isolates reflected the broad distribution and variety of acquired β-lactamases, including those that confer resistance to third-generation cephalosporins and carbapenems. Besides, these results confirmed that the presence of acquired β-lactamases alone is insufficient to cause cefiderocol resistance. While 54.4% of isolates produced at least one β-lactamase, only 14% of isolates were resistant to cefiderocol by EUCAST breakpoints. Some of the β-lactamases previously associated with reduced cefiderocol susceptibility include class A ESBL (PER-1 or SHV-12), MBL (some NDM in Enterobacterales) or some class D enzymes, such as OXA-2 or OXA-10 ESBL variants.^11–18^ In our dataset, NDM and KPC were exclusively detected in cefiderocol-resistant isolates, albeit in a limited number of cases, and were always co-expressed with additional β-lactamases. Conversely, VIM and OXA-2, the most prevalent β-lactamases in *P. aeruginosa*, were more frequently observed in susceptible isolates than in resistant ones.

Cefiderocol resistance has been reported to involve the inactivation of iron-uptake-related genes that are crucial for the antibiotic’s siderophore-mediated entry mechanism.^12,14–16^ In our sample, five out of the eight cefiderocol-resistant isolates exhibited mutations in these genes (i.e. *cirA, tonB, piuA,* and *pirR*). These results suggest that resistance likely arises from the interplay between β-lactamase production, impaired drug uptake, and other potential resistance mechanisms. In contrast to other studies, mutations in PBP3 were absent among the resistant isolates in this collection, indicating that target alteration was not the primary resistance mechanism in these isolates.^12–14^

Overall, these observations emphasize the multifactorial nature of cefiderocol resistance and the difficulty of establishing a direct correlation between MIC values and specific β-lactamase genotypes or cefiderocol-exclusive resistance mutations, especially given the limited sample size and geographical distribution.^27^

Regarding susceptibility testing for cefiderocol, BMD in iron-depleted media is the current reference method according to EUCAST criteria, although it faces several limitations.^22^ Its technical complexity, mainly due to medium preparation, significantly hinders its implementation in routine laboratory practice.^22^ Consequently, alternative standardized commercial BMD devices (UMIC^®^ and ComASP^®^), which already include iron-depleted cation-adjusted Mueller–Hinton broth, have been developed. These methods have demonstrated adequate concordance with reference susceptibility testing approaches.^20,21,23,25,26,39^ As such, they represent practical and potentially reliable options for use in clinical laboratories. Nevertheless, EUCAST validation and endorsement are still pending.

In this study, UMIC^®^ and ComASP^®^ showed good results, meeting the performance criteria of ISO 20776-2.^40^ CA and EA were above 90% in all cases, with no very major errors and only one major error for UMIC^®^. In contrast, MIC strips and DD methods showed poorer results with our isolates, with two very major errors in each case caused by the same two isolates. Moreover, the interpretation of ATUs in DD methods remains problematic.^23,24^ In the absence of a retest following EUCAST recommendations, laboratories often report ATU as resistant, which significantly increases the report of false resistant isolates, negatively influencing confidence in cefiderocol. Establishing reliable methods to test cefiderocol susceptibility is essential to avoid unnecessarily limiting therapeutic options for critically ill patients. Therefore, commercial BMD methods may represent a viable alternative to other commercial alternatives and the current reference method for routine cefiderocol susceptibility testing in clinical laboratories.

Overall clinical cure and mortality rates in this subset of patients were similar to the results in the full cohort.^27^ Also, despite the low number of patients in the Enterobacterales group, both this and the *P. aeruginosa* group showed similar clinical outcomes. Despite eight isolates exhibiting MICs above 2 mg/L (three isolates with MIC of 4 mg/L and five with MIC >4 mg/L), considered resistant by EUCAST breakpoints, clinical outcomes were generally favourable, with seven of these patients achieving clinical cure. This discrepancy may be partially explained by the discordance in susceptibility breakpoints between EUCAST (≤2 mg/L) and CLSI (≤4 mg/L).^24,41^ It is possible that patients infected by isolates with elevated cefiderocol MIC values had plasma concentrations above the MIC values during cefiderocol treatment, which resulted in favourable outcome; however, plasma concentrations are not available. In a separate study in patients treated with cefiderocol under the compassionate use programme, a small number of patients (*n* = 4) had infections caused by *P. aeruginosa* isolates with increased MIC values (i.e. MIC ≥8 mg/L) by BMD method and responded to cefiderocol treatment.^41^ Although, in this study, the categories of susceptibility were interpreted by FDA and CLSI breakpoints.^41^ Furthermore, *in vivo* factors, such as patient-specific drug distribution, infection site, local microenvironment, and bacterial gene expression dynamics, as well as other clinical factors, such as previous infection management, source control when appropriate, and combination therapy, likely influence treatment failure or success but require further exploration to fully elucidate their roles.

Our study has some limitations in the comparison of microbiological data and clinical outcomes. First, cefiderocol treatment was selected without report of susceptibility to this agent and it was prescribed by physicians when no other treatment options/alternatives were available. An additional limitation was that clinical outcome was recorded for all patients in the PERSEUS study, however microbiological cure was not reported.^27,28^ Despite this fact, our study reflects real-life cases and later use of the standard ISO BMD method using iron-depleted cation-adjusted MH broth for comparison of different methods.

In conclusion, cefiderocol shows great potential as a therapeutic option against MDR Gram-negative bacterial infections, especially those involving *P. aeruginosa*. Resistance mechanisms require further investigation but likely involve a combination of factors, including β-lactamase production and impaired antibiotic uptake via iron transport pathways. Validating and standardizing practical susceptibility testing methods will be pivotal in optimizing cefiderocol clinical use. Moreover, future studies investigating MIC breakpoints in relation to clinical outcomes, as well as *in vivo* pharmacodynamics, are essential for the adoption of cefiderocol, particularly for critically ill patients with limited treatment options.

## Supplementary Material

dkag164_Supplementary_Data

## Supplementary data

Tables S1 to S6 is available as Supplementary data at *JAC* Online.

## References

[1] WHO . WHO Bacterial Priority Pathogen List, 2024: bacterial pathogens of public health importance to guide research, development and strategies to prevent and control antimicrobial resistance. 2024. https://iris.who.int/bitstream/handle/10665/376776/9789240093461-eng.pdf?sequence=1. 10.1016/S1473-3099(25)00118-5 PMC12367593 40245910

[2] Murray CJ, Ikuta KS, Sharara F et al Global burden of bacterial antimicrobial resistance in 2019: a systematic analysis. Lancet 2022; 399: 629–55. 10.1016/S0140-6736(21)02724-0 35065702 PMC8841637

[3] Sato T, Yamawaki K. Cefiderocol: discovery, chemistry, and *in vivo* profiles of a novel siderophore cephalosporin. Clin Infect Dis 2019; 69: S538–43. 10.1093/cid/ciz826 31724047 PMC6853759

[4] Committee for Medicinal Products for Human Use (CHMP) . European Medicines Agency. *Summary of Product Characteristics Fetcroja*. https://www.ema.europa.eu/en/documents/product-information/fetcroja-epar-product-information_en.pdf.

[5] Ito-Horiyama T, Ishii Y, Ito A et al Stability of novel siderophore cephalosporin S-649266 against clinically relevant carbapenemases. Antimicrob Agents Chemother 2016; 60: 4384–6. 10.1128/AAC.03098-15 27139465 PMC4914688

[6] Poirel L, Kieffer N, Nordmann P. Stability of cefiderocol against clinically significant broad-spectrum oxacillinases. Int J Antimicrob Agents 2018; 52: 866–7. 10.1016/j.ijantimicag.2018.11.005 30415004

[7] Ito A, Nishikawa T, Ota M et al Stability and low induction propensity of cefiderocol against chromosomal AmpC β-lactamases of *Pseudomonas aeruginosa* and *Enterobacter cloacae*. J Antimicrob Chemother 2018; 73: 3049–52. 10.1093/jac/dky317 30188999 PMC6198743

[8] Bassetti M, Echols R, Matsunaga Y et al Efficacy and safety of cefiderocol or best available therapy for the treatment of serious infections caused by carbapenem-resistant Gram-negative bacteria (CREDIBLE-CR): a randomised, open-label, multicentre, pathogen-focused, descriptive, phase 3 trial. Lancet Infect Dis 2021; 21: 226–40. 10.1016/S1473-3099(20)30796-9 33058795

[9] Ito A, Sato T, Ota M et al *In vitro* antibacterial properties of cefiderocol, a novel siderophore cephalosporin, against gram-negative bacteria. Antimicrob Agents Chemother 2018; 62: e01454-17. 10.1128/AAC.01454-17 PMC5740388 29061741

[10] Kimbrough JH, Maher JM, Sader HS et al *In vitro* activity assessment of cefiderocol against *Enterobacterales, Pseudomonas aeruginosa*, and *Acinetobacter* spp., including β-lactam nonsusceptible molecularly characterized isolates, collected from 2020 to 2021 in the United States and European hospitals. Microbiol Spectr 2024; 12: e0147424. 10.1128/spectrum.01474-24 39387599 PMC11537082

[11] Takemura M, Wise MG, Hackel MA et al *In vitro* activity of cefiderocol against MBL-producing Gram-negative bacteria collected in North America and Europe in five consecutive annual multinational SIDERO-WT surveillance studies (2014-2019). J Antimicrob Chemother 2023; 78: 2019–27. 10.1093/jac/dkad200 37390312 PMC10393876

[12] Maruri-Aransolo A, López-Causapé C, Hernández-García M et al *In vitro* activity of cefiderocol in *Pseudomonas aeruginosa* isolates from people with cystic fibrosis recovered during three multicentre studies in Spain. J Antimicrob Chemother 2024; 79: 1432–40. 10.1093/jac/dkae126 38708553

[13] Lasarte-Monterrubio C, Fraile-Ribot PA, Vázquez-Ucha JC et al Activity of cefiderocol, imipenem/relebactam, cefepime/taniborbactam and cefepime/zidebactam against ceftolozane/tazobactam and ceftazidime/avibactam-resistant *Pseudomonas aeruginosa*. J Antimicrob Chemother 2022; 77: 2809–15. 10.1093/jac/dkac241 35904000

[14] Gomis-Font MA, Sastre-Femenia M, Taltavull B et al *In vitro* dynamics and mechanisms of cefiderocol resistance development in wild-type, mutator and XDR *Pseudomonas aeruginosa*. J Antimicrob Chemother 2023; 78: 1785–94. 10.1093/jac/dkad172 37253034

[15] Karakonstantis S, Rousaki M, Vassilopoulou L et al Global prevalence of cefiderocol non-susceptibility in *Enterobacterales, Pseudomonas aeruginosa, Acinetobacter baumannii*, and *Stenotrophomonas maltophilia*: a systematic review and meta-analysis. Clin Microbiol Infect 2024; 30: 178–88. 10.1016/j.cmi.2023.08.029 37666449

[16] Candel FJ, Santerre Henriksen A, Longshaw C et al *In vitro* activity of the novel siderophore cephalosporin, cefiderocol, in Gram-negative pathogens in Europe by site of infection. Clin Microbiol Infect 2022; 28: 447.e1–e6. 10.1016/j.cmi.2021.07.018 34298176

[17] Fröhlich C, Sørum V, Tokuriki N et al Evolution of β-lactamase-mediated cefiderocol resistance. J Antimicrob Chemother 2022; 77: 2429–36. 10.1093/jac/dkac221 35815680 PMC9410664

[18] Delgado-Valverde M, Portillo-Calderón I, Recacha E et al *In vitro* activity of cefiderocol compared to other antimicrobials against a collection of metallo-beta-lactamase-producing Gram-negative bacilli from southern Spain. Microbiol Spectr 2023; 11: e0493622. 10.1128/spectrum.04936-22 37249425 PMC10269457

[19] Santerre Henriksen A, Jeannot K, Oliver A et al In vitro activity of cefiderocol against European *Pseudomonas aeruginosa* and *Acinetobacter* spp., including isolates resistant to meropenem and recent β-lactam/β-lactamase inhibitor combinations. Microbiol Spectr 2024; 12: e0383623. 10.1128/spectrum.03836-23 38483164 PMC10986614

[20] Emeraud C, Gonzalez C, Dortet L. Comparison of ComASP^®^ and UMIC^®^ methods with the reference method for cefiderocol susceptibility testing on carbapenem-resistant *Enterobacterales*. J Antimicrob Chemother 2023; 78: 1800–1. 10.1093/jac/dkad134 37141286

[21] Bianco G, Boattini M, Comini S et al Disc diffusion and ComASP^®^ cefiderocol microdilution panel to overcome the challenge of cefiderocol susceptibility testing in clinical laboratory routine. Antibiotics (Basel) 2023; 12: 604. 10.3390/antibiotics12030604 36978470 PMC10045311

[22] EUCAST . EUCAST guidance document on broth microdilution testing of cefiderocol. https://www.eucast.org/fileadmin/src/media/PDFs/EUCAST_files/Guidance_documents/Cefiderocol_MIC_testing_EUCAST_guidance_document_January_2024.pdf.

[23] EUCAST . EUCAST warnings concerning antimicrobial susceptibility testing products or procedures. https://www.eucast.org/ast-of-bacteria/warnings.

[24] EUCAST . Breakpoint tables for interpretation of MICs and zone diameters. Version 15.0. 2025. https://www.eucast.org/fileadmin/src/media/PDFs/EUCAST_files/Breakpoint_tables/v_15.0_Breakpoint_Tables.pdf.

[25] Stracquadanio S, Nicolosi A, Marino A et al Issues with cefiderocol testing: comparing commercial methods to broth microdilution in iron-depleted medium—analyses of the performances, ATU, and trailing effect according to EUCAST initial and revised interpretation criteria. Diagnostics (Basel) 2024; 14: 2318. 10.3390/diagnostics14202318 39451641 PMC11506871

[26] Dortet L, Niccolai C, Pfennigwerth N et al Performance evaluation of the UMIC^®^ cefiderocol to determine MIC in Gram-negative bacteria. J Antimicrob Chemother 2023; 78: 1672–6. 10.1093/jac/dkad149 37209112 PMC10320108

[27] Torre-Cisneros J, Almirante B, Martos CF et al Effectiveness and safety of cefiderocol treatment in patients with Gram-negative bacterial infections in Spain in the early access programme: results of the PERSEUS study. Eur J Clin Microbiol Infect Dis 2025; 44: 1375–90. 10.1007/s10096-025-05108-6 40131647 PMC12116923

[28] Torre-Cisneros J, Ferrer R, Martos CF et al Cefiderocol treatment for patients infected by *Stenotrophomonas maltophilia*, *Burkholderia cepacia* complex and *Achromobacter* spp.: subgroup analysis from the PERSEUS study. Eur J Clin Microbiol Infect Dis 2025; 44: 1367–74. 10.1007/s10096-025-05109-5 40126766 PMC12116654

[29] International Organization for Standarization (ISO) . *ISO 20776-1:2019.* Susceptibility testing of infectious agents and evaluation of performance of antimicrobial susceptibility test devices Part 1: Broth micro-dilution reference method for testing the in vitro activity of antimicrobial agents against rapidly growing aerobic bacteria involved in infectious diseases. 2019. https://www.iso.org/standard/70464.html

[30] EUCAST . Breakpoint tables for interpretation of MICs and zone diameters. Version 14.0. 2024. https://www.eucast.org/fileadmin/src/media/PDFs/EUCAST_files/Breakpoint_tables/v_14.0_Breakpoint_Tables.pdf.

[31] CLSI . *Performance Standards for Antimicrobial Susceptibility Testing*: *M100. 2024*.

[32] Bankevich A, Nurk S, Antipov D et al SPAdes: a new genome assembly algorithm and its applications to single-cell sequencing. J Comput Biol 2012; 19: 455–77. 10.1089/cmb.2012.0021 22506599 PMC3342519

[33] Zankari E, Hasman H, Cosentino S et al Identification of acquired antimicrobial resistance genes. J Antimicrob Chemother 2012; 67: 2640–4. 10.1093/jac/dks261 22782487 PMC3468078

[34] Sastre-Femenia MÀ, Fernández-Muñoz A, Gomis-Font MA et al *Pseudomonas aeruginosa* antibiotic susceptibility profiles, genomic epidemiology and resistance mechanisms: a nation-wide five-year time lapse analysis. Lancet Reg Health Eur 2023; 34: 100736. 10.1016/j.lanepe.2023.100736 37753216 PMC10518487

[35] Maraolo AE, Licciardi F, Gentile I et al *Stenotrophomonas maltophilia* infections: a systematic review and meta-analysis of comparative efficacy of available treatments, with critical assessment of novel therapeutic options. Antibiotics (Basel) 2023; 12: 910. 10.3390/antibiotics12050910 37237813 PMC10215754

[36] Gill CM, Santini D, Nicolau DP. In vitro activity of cefiderocol against a global collection of carbapenem-resistant Pseudomonas aeruginosa with a high level of carbapenemase diversity. J Antimicrob Chemother 2024; 79: 412–6. 10.1093/jac/dkad396 38153232 PMC10832583

[37] Ordooei Javan A, Shokouhi S, Sahraei Z. A review on colistin nephrotoxicity. Eur J Clin Pharmacol 2015; 71: 801–10. 10.1007/s00228-015-1865-4 26008213

[38] Eljaaly K, Bidell MR, Gandhi RG et al Colistin nephrotoxicity: meta-analysis of randomized controlled trials. Open Forum Infect Dis 2021; 8: ofab026. 10.1093/ofid/ofab026 33623807 PMC7888569

[39] Irigoyen-Von-Sierakowski A, Ocaña A, Sánchez-Mayoral R et al Real-world performance of susceptibility testing for cefiderocol: insights from a prospective multicentre study on Gram-negative bacteria. JAC Antimicrob Resist 2024; 6: dlae169. 10.1093/jacamr/dlae169 39464858 PMC11503648

[40] International Organization for Standarization (ISO) . ISO 20776-2:2021. Clinical laboratory testing and in vitro diagnostic test systems — Susceptibility testing of infectious agents and evaluation of performance of antimicrobial susceptibility test devices Part 2: Evaluation of performance of antimicrobial susceptibility test devices against reference broth micro-dilution. 2021. https://www.iso.org/standard/79377.html

[41] Satlin MJ, Simner PJ, Slover CM et al Cefiderocol treatment for patients with multidrug- and carbapenem-resistant *Pseudomonas aeruginosa* infections in the compassionate use program. Antimicrob Agents Chemother 2023; 67: e0019423. 10.1128/aac.00194-23 37347188 PMC10353454

