# Supplementary material for: Cefiderocol activity against multidrug-resistant isolates from the PERSEUS study: relationship between cefiderocol minimum inhibitory concentration and clinical outcomes
Source: J Antimicrob Chemother. 2026 May 26;81(6):dkag164. doi: 10.1093/jac/dkag164 (PMC13201092; doi:10.1093/jac/dkag164)
Supplement: dkag164_Supplementary_Data [file dkag164_supplementary_data.zip › Canton_PERSEUS_Micro_Supplementary-revision-clean-submission.docx]

**Supplementary data**

**Cefiderocol activity against multidrug-resistant isolates from the PERSEUS study: relationship between cefiderocol minimum inhibitory concentration and clinical outcomes**

**Rafael Cantón**^1,2^**, María García-Castillo**^1,2^**, Marta Hernández-García**^1,2^**, Miquel Àngel Sastre-Femenía**^2,3^**, Carla López-Causapé**^2,3^**, Patricia Ruiz-Garbajosa**^1,2^**, Lucía García Labrador^4*^, Christopher Longshaw^5^, Antonio Oliver**^2,3^

^1^Servicio de Microbiología Clínica, Hospital Universitario Ramón y Cajal and Instituto Ramón y Cajal de Investigación Sanitaria (IRYCIS), Madrid, Spain.

^2^CIBER de Enfermedades Infecciosas (CIBERINFEC), Instituto de Salud Carlos III, Madrid, Spain.

^3^Servicio de Microbiología, Hospital Universitario Son Espases, Instituto de Investigación Sanitaria Illes Balears (IdISBa), Palma de Mallorca, Spain.

^4^Medical Affairs, Shionogi S.L.U., Madrid.

^5^Medical Affairs, Shionogi B.V., London, United Kingdom.

*Corresponding author: Lucía García Labrador

Adress: Shionogi S.L.U, C/Serrano 45, Madrid, Spain

e-mail: [lucia.garcia@shionogi.eu](mailto:lucia.garcia@shionogi.eu)

**Supplementary Table S1.** Antimicrobials and tested concentration ranges

| **Antimicrobial** | **MIC range (mg/L)** |
| --- | --- |
| Aztreonam (AZT) | 1-32 |
| Piperacillin/Tazobactam (PT4) | 4-32 |
| Cefepime (FEP) | 1-16 |
| Ceftazidime-avibactam (CZA) | 0.25-16 |
| Ceftolozane-tazobactam (CT) | 0.25-8 |
| Imipenem (IMI) | 1-8 |
| Imipenem-Relebactam (IMR) | 0.06-8 |
| Meropenem (MERO) | 0.12-16 |
| Meropenem-vaborbactam (MEV) | 0.06-16 |
| Tigecycline (TGC) | 0.5-1 |
| Eravacycline (ERV) | 0.008-0.5 |
| Amikacin (AMI) | 2-32 |
| Tobramycin (TOB) | 0.5-4 |
| Fosfomycin (FOS) | 16-64 |
| Colistin (COL) | 0.5-16 |
| Cefiderocol (FDC)* | ≤0.12->16 |
| *ISO broth microdilution following EUCAST recommendations. | |

**Supplementary Table S2.** MIC_50_, MIC_90_ and susceptibility of 43 *Pseudomonas* spp. and 8 *Enterobacterales* isolates to cefiderocol and comparators

| **Antimicrobial** | **MIC (mg/L)** | | | **EUCAST  (nº isolates)** | | | **EUCAST  (%)** | | |
| --- | --- | --- | --- | --- | --- | --- | --- | --- | --- |
|  | **MIC 50%** | **MIC 90%** | **Range** | **S** | **I** | **R** | **S** | **I** | **R** |
| Aztreonam | >32 | >32 | 1-32 | 0 | 14 | 37 | 0.0 | 27.5 | 72.5 |
| Piperacillin/Tazobactam | >32/4 | >32/4 | 4-32 | 0 | 1 | 50 | 0.0 | 2.0 | 98.0 |
| Cefepime | >16 | >16 | 1-16 | 0 | 1 | 50 | 0.0 | 2.0 | 98.0 |
| Ceftazidime-avibactam | >16/4 | >16/4 | 0.25-16 | 9 | 0 | 42 | 17.6 | 0.0 | 82.4 |
| Ceftolozane-tazobactam | >8/4 | >8/4 | 0. 25-8 | 11 | 0 | 40 | 21.6 | 0.0 | 78.4 |
| Imipenem | >8 | >8 | 1-8 | 0 | 3 | 48 | 0.0 | 5.9 | 94.1 |
| Imipenem-Relebactam | >8/4 | >8/4 | 0.06-8 | 5 | 0 | 46 | 9.8 | 0.0 | 90.2 |
| Meropenem | >16 | >16 | 0.12-16 | 2 | 0 | 49 | 3.9 | 0.0 | 96.1 |
| Meropenem-vaborbactam | >16/8 | >16/8 | 0.06-16 | 7 | 0 | 44 | 13.7 | 0.0 | 86.3 |
| Tigecycline | >1 | >1 | 0.5-1 | - | - | - | - | - | - |
| Eravacycline | >0.5 | >0.5 | 0.008-0.5 | 1 | 0 | 7 | 12.5 | 0.0 | 87.5 |
| Amikacin | 16 | >32 | 2-32 | 32 | 0 | 19 | 62.7 | 0.0 | 37.3 |
| Tobramycin | >4 | >4 | 0.5-4 | 14 | 0 | 37 | 27.5 | 0.0 | 72.5 |
| Fosfomycin | >64 | >64 | 16-64 | - | - | - | - | - | - |
| Colistin | 1 | 4 | 0.5-16 | 50 | 0 | 1 | 98.0 | 0.0 | 2.0 |
| Cefiderocol | 0.5 | 4 | ≤0.12->16 | 43 | 0 | 8 | 84.3 | 0.0 | 15.7 |

S, Sensitive; I, Susceptible, increased exposure; R, Resistant

**Supplementary Table S3.** MIC_50_, MIC_90_ and susceptibility of 41 *P. aeruginosa* isolates to cefiderocol and comparators

| **Antimicrobial** | **MIC (mg/L)** | | | **EUCAST  (nº isolates)** | | | **EUCAST  (%)** | | |
| --- | --- | --- | --- | --- | --- | --- | --- | --- | --- |
|  | **MIC 50%** | **MIC 90%** | **Range** | **S** | **I** | **R** | **S** | **I** | **R** |
| Aztreonam | 32 | >32 | 1-32 | 0 | 13 | 28 | 0.0 | 31.7 | 68.3 |
| Piperacillin/Tazobactam | >32/4 | >32/4 | 4-32 | 0 | 1 | 40 | 0.0 | 2.4 | 97.6 |
| Cefepime | >16 | >16 | 1-16 | 0 | 1 | 40 | 0.0 | 2.4 | 97.6 |
| Ceftazidime-avibactam | >16/4 | >16/4 | 0.25-16 | 8 | 0 | 33 | 19.5 | 0.0 | 80.5 |
| Ceftolozane-tazobactam | >8/4 | >8/4 | 0. 25-8 | 11 | 0 | 30 | 26.8 | 0.0 | 73.2 |
| Imipenem | >8 | >8 | 1-8 | 0 | 3 | 38 | 0.0 | 7.3 | 92.7 |
| Imipenem-Relebactam | >8/4 | >8/4 | 0.06-8 | 4 | 0 | 37 | 9.8 | 0.0 | 90.2 |
| Meropenem | >16 | >16 | 0.12-16 | 2 | 0 | 39 | 4.9 | 0.0 | 95.1 |
| Meropenem-vaborbactam | >16/8 | >16/8 | 0.06-16 | 6 | 0 | 35 | 14.6 | 0.0 | 85.4 |
| Tigecycline | >1 | >1 | 0.5-1 | - | - | - | - | - | - |
| Eravacycline | >0.5 | >0.5 | 0.008-0.5 | - | - | - | - | - | - |
| Amikacin | 16 | >32 | 2-32 | 29 | 0 | 12 | 70.7 | 0.0 | 29.3 |
| Tobramycin | >4 | >4 | 0.5-4 | 14 | 0 | 27 | 34.1 | 0.0 | 65.9 |
| Fosfomycin | >64 | >64 | 16-64 | - | - | - | - | - | - |
| Colistin | 1 | 2 | 0.5-16 | 40 | 0 | 1 | 97.6 | 0.0 | 2.4 |
| Cefiderocol | 0.5 | 2 | ≤0.12->16 | 38 | 0 | 3 | 92.7 | 0.0 | 7.3 |

S, Sensitive; I, Susceptible, increased exposure; R, Resistant

**Supplementary Table S5.** Prevalence of acquired β-lactamases among 43 *Pseudomonas* spp. and 8 *Enterobacterales* isolates according to Ambler classification criteria

| **Type of β-lactamase** | ***Pseudomonas* spp. with acquired β-lactamases**  **(n = 23)** | | **Enterobacterales with acquired β-lactamases**  **(n = 8)** | |
| --- | --- | --- | --- | --- |
|  | **% (n)** | **β-lactamase (n)** | **% (n)** | **β-lactamase (n)** |
| A | 17.4% (4) | CARB-4 (E203K) (3); GES-7 (1) | 75% (6) | CTX-M-15 (3); TEM-1B (2); TEM-1A (1); KPC-3 (1); SHV-12 (1) |
| B | 78.3 (18) | VIM-2 (11); VIM-1 (4); VIM-20 (2); IMP-15 (1) | 50% (4) | NDM-1 (3); VIM-1 (2) |
| C | 4.3% (1) | FOX-4 (H220Y, V233A) (1) | 25% (2) | CMY-4 (1); DHA-1 (1) |
| D | 39.1% (9) | OXA-2 (6); OXA-1372 (1); OXA-210 (1); OXA-4 (1) | 62.5% (5) | OXA-1 (2); OXA-48 (2); OXA-9 (W112X) (1) |

CARB, carbenicillin-hydrolysing β-lactamase; CMY, plasmid-mediated cephamycin-hydrolysing AmpC β-lactamase; CTX-M; cefotaximase Munchen extended-spectrum β-lactamase; DHA, plasmid-mediated inducible AmpC β-lactamase; FOX, plasmid-mediated cefoxitin-hydrolysing AmpC β-lactamase; GES, Guiana extended-spectrum β-lactamase; IMP, imipenemase metallo-β-lactamase; KPC, *Klebsiella pneumoniae* carbapenemase; NDM, New Delhi metallo-β-lactamase; OXA, oxacillinase; SHV, sulfhydryl variable extended-spectrum β-lactamase; TEM, Temoneira extended-spectrum β-lactamase; VIM, Verona integron-encoded metallo-β-lactamase.
